# Acetamiprid Exposure Disrupts Gut Microbiota in Adult and Larval Worker Honeybees (*Apis mellifera* L.)

**DOI:** 10.3390/insects15120927

**Published:** 2024-11-26

**Authors:** Yuchen Su, Jingliang Shi, Yueyang Hu, Jianhui Liu, Xiaobo Wu

**Affiliations:** 1 Honeybee Research Institute, Jiangxi Agricultural University, Nanchang 330045, China; mtdz900828@163.com (Y.S.); sjl_net@163.com (J.S.); hu_yueyang@163.com (Y.H.); liujh0507@163.com (J.L.); 2 Jiangxi Province Key Laboratory of Honeybee Biology and Beekeeping, Nanchang 330045, China

**Keywords:** honeybees, acetamiprid, gut microbiota, 16S rRNA

## Abstract

Acetamiprid is a third-generation neonicotinoid insecticide that is now widely employed for the protection of crops grown in outdoor environments. This is because it is considerably less toxic to pollinating insects than other neonicotinoids. Previous studies have shown that acetamiprid has direct physiological effects on adult and larval bees. However, its effects on the potentially healthy gut microbiota of honeybees have not been fully elucidated. To further investigate the effects, adult and larval worker honeybees were exposed to sucrose solutions containing acetamiprid at concentrations of 0, 5, and 25 mg/L for a period of 7 days (adults) and 4 days (larvae). The results showed that acetamiprid exposure significantly disrupted the honeybees’ intestinal microbiota. In adults, acetamiprid exposure led to a significant increase in the relative abundance of *Commensalibacter*, while the *Bifidobacterium* and *Gilliamella* levels decreased. In larvae, we observed significant changes in the microbial composition, notably a marked reduction in *Bombella*. Further analysis demonstrated that alterations in the gut microbiota of honeybee larvae were associated with disturbances in metabolic pathways that regulate energy metabolism and neurometabolism. These results suggest that acetamiprid affects bee health not only through direct physiological effects, but also through changes in the gut microbiota, which in turn affect the metabolic and immune function of bees. This study underscores the need to evaluate pesticides’ risks from a microbiological standpoint and offers crucial insights into how acetamiprid impacts bee health by modifying the gut microbiota. These insights support the more comprehensive assessment of acetamiprid and similar pesticides regarding bee health.

## Simple Summary

The pesticide acetamiprid, widely used to protect outdoor crops, poses lesser risks to pollinators like honeybees compared to other pesticides. However, its impact on honeybees’ gut microbiota remains unclear. This study revealed that acetamiprid significantly altered the gut microbial community in both adult and larval bees, leading to disturbances in energy and neurometabolic pathways. This indicates that acetamiprid affects bee health not only directly but also indirectly through changes in the gut microbiota. Hence, the assessment of pesticides’ risks must consider their effects on bee gut bacteria. This study provides crucial insights for a comprehensive understanding of acetamiprid’s impact on bee health.

## 1. Introduction

Honeybees (*Apis mellifera*) are one of the most important pollinator species in natural ecosystems and agricultural production [1,2,3]. The declining trend in global honeybee populations in recent years will pose a major challenge to agricultural production and environmental ecology [4,5]. There are many factors contributing to the declining trend of honeybee populations, such as mites, viruses, climate change, pesticide misuse, and environmental pollution [6,7,8]. One of the main causes is the damage to honeybee colonies due to the misuse of pesticides [9,10]. Honeybees are inevitably exposed to pesticides during the pollination process, which have lethal or sublethal effects on bees, resulting in a reduced pollination capacity and colony losses [11,12].

Neonicotinoid pesticides are widely used insecticides worldwide and once accounted for one-quarter of the insecticide market share [13,14]. They work by disrupting nerve signaling in the insect central nervous system, significantly impairing neural function [15,16,17,18]. This disruption has been identified as a major factor in colony size reduction and decreased overwintering success [19,20]. The European Union has restricted the use of some neonicotinoid insecticides in recent years, including imidacloprid (EU, 2018/783), clothianidin (EU, 2018/784), thiamethoxam (EU, 2018/785), and thiacloprid (EU, 2020/23), leading to a significant reduction in the use of these pesticides. In addition to emergency approvals for some neonicotinoids, only acetamiprid is currently approved for outdoor use, which has been extended until 2033 (EU, 2018/113) [21]. Acetamiprid is a safe and low-toxicity insecticide. As a third-generation neonicotinoid belonging to the cyano-substituted neonicotinoids, it is much safer than nitro-substituted neonicotinoids such as imidacloprid, thiamethoxam, and clothianidin. This has led to its widespread use in agriculture in recent years [22,23]. However, the assessment of the risk of a pesticide to bees should focus more on its sublethal effects and potential biological effects, as opposed to acute toxicity, where bees are usually exposed to and affected by sublethal doses of pesticides. At actual environmental concentrations of 50–500 mg/L of acetamiprid residues, sublethal doses of acetamiprid have been shown to affect the expression of learning and memory-related genes in workers, leading to a significant reduction in memory capacity, and to affect juvenile hormone synthesis and utilization proteins in queens [24]. Acetamiprid above 5 mg/L had a significant effect on the birth weight and emerging rates of worker bees, and acetamiprid above 25 mg/L reduced the larval capping rate and significantly reduced the lifespan of workers [25]. Furthermore, a metabolomic analysis of honeybee larvae exposed to sublethal doses revealed that acetamiprid solution residues in bee food exceeding 5 mg/L resulted in metabolic disorders in honeybee larvae [26]. It is not only the effects on bee longevity, behavior, and physiology that are of concern; other sublethal effects are also important—a viewpoint that is increasingly being taken into consideration [27]. In fact, the gut microbiota plays a significant role in helping bees to resist exogenous contaminants and has been established as an effective biological indicator for environmental detection [28]. However, further research is needed to determine the effect of acetamiprid exposure on the gut microbiota structure in adults and larvae.

Recent years have seen a growing focus among researchers on the impact of stress on the gut microbiota and the resulting interactions with the host [29,30,31]. While most previous studies on the effects of pesticides on honeybee health have focused on direct effects on various physiological indicators in honeybees, there is growing evidence that such effects extend to the honeybee gut microbiota. The gut microbiota is interconnected to multiple aspects of host function through metabolism, proteins, and genes, and it has emerged as a key factor in evaluating and studying bee health in relation to the potential effects of pesticides [32]. The honeybee gut microbiome is relatively simple and conservative, with 95–99% of the gut microbes dominated by a core phylotype of eight species in worker bees [33,34,35]. However, it has indispensable effects on honeybees in terms of weight gain, immune detoxification, and social behavior [36,37,38,39]. A healthy gut microbiota is a fundamental indicator of colony health [40]. In the future, monitoring the gut microbiota of honeybees may serve as a method to assess colony health, revealing potential impacts from external pollutants or viruses. Many studies definitively show that various insecticides have significant effects on the gut microbiota of both adults and larvae, leading to impaired bee health [29,40,41,42,43,44]. Meanwhile, the correlational analysis of changes in the gut microbiota and changes in host metabolism allows a more comprehensive understanding of the effects of stressors on honeybee health, as well as speculation on the manner in which the gut microbiota and host interact [45].

This study aimed to investigate the impacts of environmentally relevant concentrations of acetamiprid on the gut microbiota of adult and larval worker bees of *Apis mellifera*. Furthermore, we conducted correlation analyses to ascertain the relationship between alterations in the gut microbiota and the metabolomes of larvae following acetamiprid exposure, which was reported previously. Our research provides definitive insights into the risks that acetamiprid poses to bees and how such insecticides affect bees’ health and gut microbiota. This research highlights the need to enhance the sensitivity of environmental monitoring for insecticides, with the aim of expanding the focus beyond direct physiological toxicity to address broader potential negative impacts.

## 2. Materials and Methods

### 2.1. Honeybee Rearing

The experimental bee colonies (*Apis mellifera* L.), with similar populations, were provided by the apiary of the Honeybee Research Institute of Jiangxi Agricultural University, Nanchang, China (28.77° N, 115.83° E). The selected three experimental colonies were observed to be in good health and not threatened by pathogens (foulbrood), parasitic mites, or previous exposure to pesticides. These colonies with analogous population sizes were maintained in accordance with the established standards for rearing methods [24].

Capped brood combs with late pupae were removed from the colonies and placed in an incubator mimicking colony conditions (darkness, T 34 ± 1 °C, RH 60 ± 5%). Following a 12 h incubation period in the incubator, newly emerged worker bees were transferred to 500 mL plastic cups for rearing. Each treatment group comprised 120 bees, with four replicates (cups) per group. Furthermore, we artificially divided a new beehive foundation into three equal areas and placed it in the hive for hive construction. Then, the queen was kept to lay eggs in the empty hive for 12 h. Following a period of approximately 96 h, the eggs were incubated and developed into 2-day-old larvae for the experiment. Reference was made to the feeding method in [42].

### 2.2. Chemicals and Reagents

The acetamiprid (70% water-dispersible granules) was provided by Jiangxi Heyi Chemical Co., Ltd. (Jiujiang, China), and had high water solubility and permeability. The composition of the formulation of acetamiprid included 70% acetamiprid (main ingredient), 12% bentonite (packaging materials), 10% ammonium chloride (disintegrant), 4% ZX-D9 (dispersant), 2% naphthalenesulfonic acid–formaldehyde condensate (dispersant), and 2% M (wetting agent) [46].

Acetamiprid (active ingredient) was dissolved in a sterile 50% (*w*/*v*) sucrose solution and adjusted to the desired experimental concentration (5 and 25 mg/L) for the subsequent experiment. The prepared acetamiprid solutions were stored in a refrigerator at −4 °C and used within a week.

### 2.3. Experimental Design and Sample Collection

The experiment was designed with acetamiprid concentrations of 0, 5, and 25 mg/L, which served as the control and the relative low and high concentrations, respectively. Adults were maintained in plastic cups for daily acetamiprid exposure and feeding, and the feeding solution was renewed at the same time each day for 7 d. In the adult sampling phase, the bees were anesthetized using carbon dioxide for 10 s. Thereafter, the intestines of the worker bees were removed with sterile forceps and transferred into freezing tubes. Each tube contained a single sample from five adult guts, with four replicates for each group. In addition, the 2-day-old larvae (D2) were fed daily with experimental concentrations of acetamiprid (0, 5, and 25 mg/L). From D2 to D5, the acetamiprid solution was administered at volumes of 1.5, 2, 2.5, and 3 μL, respectively. Following a four-day feeding period, the guts of 6-day-old (prior to intestinal evacuation) larvae were collected from each group, rinsed with sterile PBS, and transferred to freezing tubes. Each tube contained a single sample derived from three larvae guts, with three replicates for each group. All sample tubes were instantly frozen in liquid nitrogen and stored at −80 °C. The gut microbes of adults who had been fed the treatment for seven days and larvae who had been fed for four days at different experimental concentrations were determined using 16S amplicon sequencing analysis.

### 2.4. Statistical Analysis

We conducted 16S rRNA sequencing and analysis based on the methodologies outlined in the literature by Lucie Kešnerová and Kasie Raymann [41,47]. A PCR reaction system was set up utilizing 30 nanograms of genomic DNA samples of satisfactory quality, along with the appropriate fusion primers. The PCR process was executed according to predetermined parameters, followed by the purification of the amplified products using magnetic beads (Agencourt AMPure XP, Beckman Coulter, Brea, CA, USA) in an elution buffer. The purified products were then labeled, and the library was constructed. The fragment size and concentration of the libraries were analyzed using an Agilent 2100 Bioanalyzer (Agilent, Santa Clara, CA, USA). Sequence splicing was conducted with the FLASH (Fast Length Adjustment of Short Reads; version 1.2.11) software. This software leverages the overlap relationship to merge paired reads from double-end sequencing into a single contiguous sequence. Subsequently, the highly variable region tags were extracted using the DADA2 (Divisive Amplicon Denoising Algorithm) within the QIIME2 software framework. By applying the DADA2 method in QIIME2, amplicon sequence variants (ASVs) were derived, where ASVs represented sequences exhibiting 100% similarity. The representative sequences of the ASVs were aligned for bacterial 16S rRNA genes using the SILVA ribosomal database (version: SSU119, http://www.arb-silva.de/, accessed on 15 July 2024).

Alpha and beta diversity analyses were performed using the QIIME software, principal coordinate analysis (PCoA), and nonparametric multidimensional scaling analysis (NMDS) based on the Bray–Curtis distance. The Kruskal test was used to compare the differences between multiple groups, and the Wilcoxon test and ANOVA were used to compare the differences between two groups. Correlation heatmap analysis was performed using Wekemo Bioincloud (https://www.bioincloud.tech, accessed on 15 July 2024) and the metabolome data pertaining to bee larvae treated with identical experimental concentrations of acetamiprid originating from a paper published by our research team [26].

## 3. Results

### 3.1. Acetamiprid Exposure Disrupts Gut Microbiota Structure in Worker Bees

**Figure 1:**
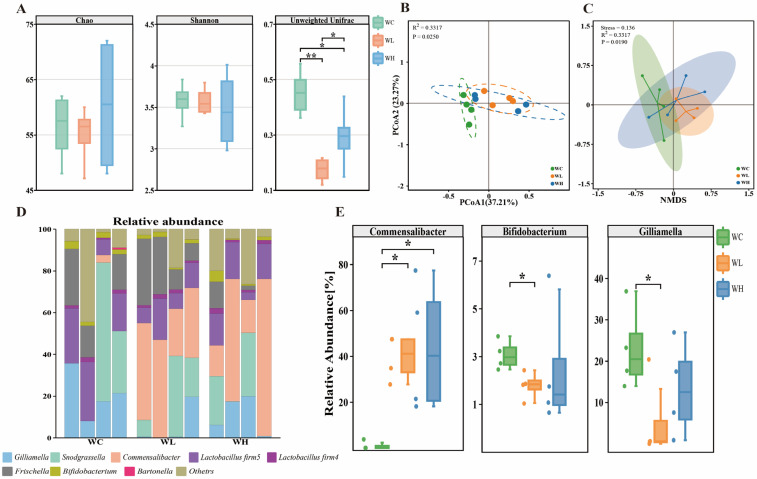
Changes in gut microbiota structure of adults after exposure to subsequent acetamiprid exposure (WH: 25 mg/L group, WL: 5 mg/L group) and control group (WC). (**A**) Differences in alpha diversity and beta diversity between different acetamiprid treatment groups. (**B**) Principal coordinate analysis (PCoA) based on Bray–Curtis distance comparisons. (**C**) Nonparametric multidimensional scale analysis (NMDS) based on Bray–Curtis distance comparisons. (**D**) The core bacterial components at the genus level. (**E**) Comparative diagram of differential bacteria of adult worker bees. (**A**,**E**) Box-and-whisker plots show high, low, and median values, with lower and upper edges of each box denoting first and third quartiles, respectively. * *p* < 0.05 and ** *p* < 0.01, Wilcoxon rank sum tests and independent-sample *t*-test. (**B**) The dashed-oval shape depicted in the image represents the 95% confidence interval.

In the 7-day-old worker bees, there were no significant differences in the alpha diversity among the three groups (Chao index: *p* = 0.76; Shannon index: *p* = 0.94) (Figure 1A), suggesting that the richness and diversity of the gut bacterial communities were not significantly affected by acetamiprid exposure. However, adults exposed to 5 and 25 mg/L of acetamiprid exhibited significant changes in their gut microbiota composition. This was evident from the unweighted UniFrac distance boxplot analysis (Wilcoxon test, *p* < 0.05) (Figure 1A), as well as the beta diversity visualizations from the principal coordinate analysis (PCoA) and nonparametric multidimensional scaling (NMDS) based on the Bray–Curtis distances (ANOSIM, *p* = 0.025, *p* = 0.019) (Figure 1B,C).

The sequencing results demonstrated that the gut bacterial community of the worker bees was constituted by eight predominant bacterial species, with the combined average relative abundance of these core species exceeding 89% of the total bacterial species. The relative abundance of *Bifidobacterium* and *Gilliamella* was found to be diminished in the acetamiprid treatment group, and the relative abundance of both core bacteria was significantly lower in the 5 mg/L acetamiprid-treated group compared to the control group. (Wilcoxon test and *t*-test, *p* < 0.05) (Figure 1E). However, no significant differences were observed between the 25 mg/L group and the other treatment groups. The abundance of *Commensalibacter* was found to be increased significantly in the gut of the honeybee following exposure to acetamiprid (Wilcoxon test, *p* < 0.05). This increase may be attributed to the bacterium’s capacity to utilize acetamiprid.

### 3.2. Acetamiprid Exposure Leads to Significant Alterations in the Structure of the Gut Microbiota of Honeybee Larvae

**Figure 2:**
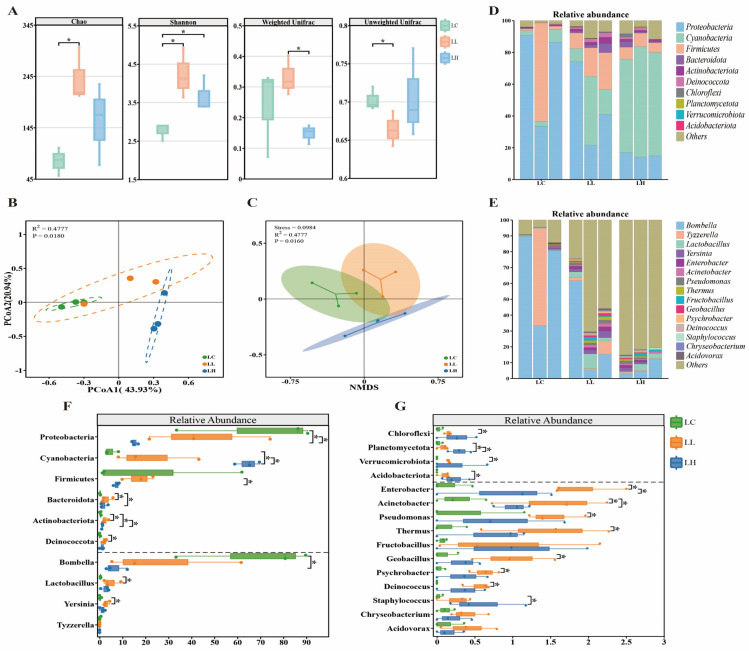
Changes in the structure of the gut microbiota of larvae exposed to acetamiprid (LH: 25 mg/L group, LL: 5 mg/L group) and control group (LC). (**A**) Differences in alpha diversity and beta diversity between different acetamiprid treatment groups. (**B**) Principal coordinate analysis (PCoA) based on Bray–Curtis distance comparisons. (**C**) Nonparametric multidimensional scale analysis (NMDS) based on Bray–Curtis distance comparisons. (**D**,**E**) Bacterial composition at phylum (**D**) and genus (**E**) level. (**F**,**G**) Comparison of bacterial differences at the phylum (above dashed line) and genus (below dashed line) levels. (**F**) Bacteria with relative abundance greater than 1%. (**G**) Bacteria with relative abundance less than 1%. (**A**,**F**,**G**) Box-and-whisker plots show high, low, and median values, with lower and upper edges of each box denoting first and third quartiles, respectively. * *p* < 0.05, Wilcoxon rank sum tests. (**B**) The dashed-oval shape depicted in the image represents the 95% confidence interval.

Additionally, we used the Chao index and Shannon index for larvae to measure the alpha diversity, reflecting changes in species diversity within the community (Figure 2A). In 6-day-old larvae, acetamiprid treatment resulted in a significant increase in alpha diversity. In particular, the Chao index and Shannon index were significantly elevated in the 5 mg/L treatment group (Wilcoxon test, *p* < 0.05). Exposure to 5 and 25 mg/L of acetamiprid significantly impacted the composition of the larval gut microbiota, as evidenced by the changes in the beta diversity. In our study, we evaluated the beta diversity through boxplot comparisons of the weighted and unweighted UniFrac distances (Wilcoxon test, *p* < 0.05) (Figure 2A), and we visualized the results using PCoA and NMDS based on the Bray–Curtis distances (Figure 2B,C). The beta diversity analysis revealed significant changes in the larval gut microbiota following acetamiprid treatment (ANOSIM, *p* = 0.018, *p* = 0.016). The microbial communities in the control group were more clustered, and there were significant differences between the control group and the two treatment groups, which further emphasized the significant deviations in the microbial composition across these groups.

Our differential analyses of the top ten phylum-level and top fifteen genus-level abundances of gut microbes in honeybee larvae clearly showed that the acetamiprid treatment had a significant impact on the structure of the microbial phylotypes (Figure 2D,E). At the phylum level, it is clear that there were significant changes in relative abundance in all ten colonies compared to the control group (Wilcoxon test, *p* < 0.05) (Figure 2F,G). Specifically, the relative abundance of *Proteobacteria* showed a significant decrease in the 5 mg/L and 25 mg/L treatment groups compared to the control group. While there was a significant difference in *Firmicutes* between the treatment groups, the decline was not statistically significant compared to the control group. Meanwhile, *Bacteroidota* and *Actinobacteriota* displayed a significant increase in relative abundance at both treatment levels, indicating a favorable response to acetamiprid exposure. Furthermore, the 5 mg/L treatment specifically promoted the growth of *Deinococcota*, *Chloroflexi*, and *Verrucomicrobiota*, whereas the 25 mg/L treatment led to a marked elevation in *Cyanobacteria*, *Planctomycetota*, and *Acidobacteriota.* At the genus level, the 5 mg/L treatment led to a significant increase in several bacterial genera, including *Lactobacillus*, *Yersinia*, *Enterobacter*, *Acinetobacter*, *Pseudomonas*, *Thermus*, *Geobacillus*, *Psychrobacter*, and *Deinococcus*. In the 25 mg/L treatment group, the significant increases in *Acinetobacter* and *Staphylococcus* were particularly prominent. Simultaneously, *Bombella*, the most abundant genus, exhibited a significant decrease, potentially creating opportunities for the proliferation of other microorganisms, resulting in an increase in the relative abundance of numerous other genera (Wilcoxon test, *p* < 0.05) (Figure 2F,G).

### 3.3. Correlation of Common Differential Metabolites of Larvae with Gut Microbiota in Combination with Acetamiprid

**Figure 3:**
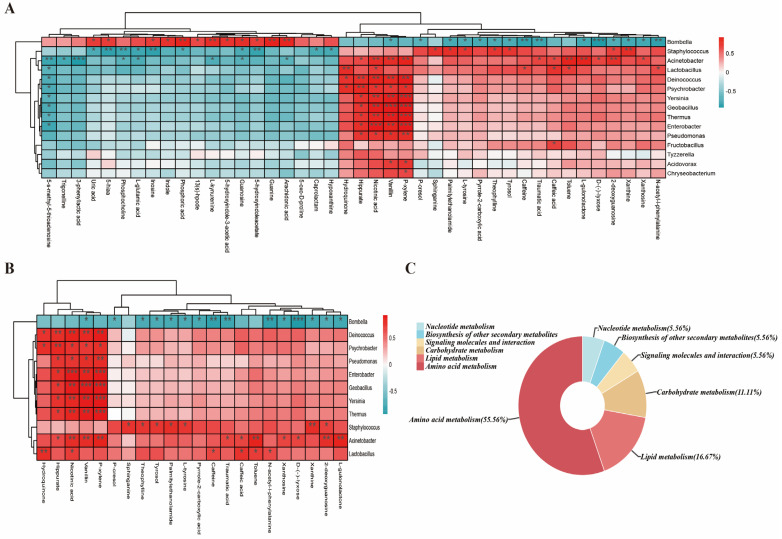
Spearman correlation analysis of gut microbiota and differential metabolites at the genus level in honeybee larvae following exposure to acetamiprid. (**A**) Heatmap of correlations between the top fifteen gut microbial and differential metabolites. (**B**) Correlation heatmap of differential bacteria and metabolites. (**C**) Metabolic pathways of metabolite aggregation of relevance to *Bombella* in the larva gut. * *p* < 0.05, ** *p* < 0.01 and *** *p* < 0.001.

A Spearman correlation analysis was conducted between the 15 most abundant genera of the honeybee larval gut microbiota and 42 common differential metabolites [26]. The results are shown in Figure 3A. This analysis revealed a significant association between the gut microbiota of the acetamiprid-exposed honeybee larvae and in vivo metabolites. It is noteworthy that eight of the eleven differential microbial genera exhibited significant positive correlations, primarily converging in four metabolites (Figure 3B). The metabolites in question were p-xylene, vanillin, nicotinic acid, and hippurate. These metabolites are intricately linked to metabolic pathways, including nicotinic acid and nicotinamide metabolism (nicotinic acid), phenylalanine metabolism (hippurate and vanillin), and the TAC energy cycle (p-xylene). These pathways are crucial for energy metabolism and detoxification processes [48,49]. This highlights the close relationship between the significantly abundant differential microbial genera and larval resistance to acetamiprid toxicity.

*Bombella* emerged as the most abundant genus in the larval gut, accounting for an average relative abundance of 67.8% in the control group, which notably declined upon acetamiprid exposure (27.4% at 5 mg/L and 6.5% at 25 mg/L) (Figure 2E). *Bombella* showed significant correlations with 27 out of 42 differential metabolites. Upon functional classification, these metabolites primarily involved amino acid metabolism (55.56%, predominant), along with lipid metabolism, carbohydrate metabolism, signaling molecules and interactions, the biosynthesis of other secondary metabolites, and nucleotide metabolism (Figure 3C). This finding reinforces the notion that *Bombella* plays a pivotal role in amino acid metabolism in larval bees, which is in line with the findings of previous research [50]. Further delving into the metabolic pathways impacted by the depletion of *Bombella*, two pivotal domains emerged as significantly disrupted: energy metabolism and neuroregulation (Table 1). In neuroregulation, the differential metabolites exhibited consistent trends, with negative correlations observed in caffeine metabolism and tyrosine metabolism and a positive correlation in tryptophan metabolism. This suggests that, under the stress induced by acetamiprid, the diminished abundance of *Bombella* may impede the elimination of excess caffeine and tyrosine, concurrently leading to a reduction in tryptophan production. These alterations may lead to perturbations in the neurometabolism of bee larvae. Additionally, the depletion of *Bombella* significantly affects phenylalanine metabolism and purine metabolism, both crucial pathways for energy production and utilization, thereby disrupting the energetic homeostasis of bee larvae. The disruption of these pathways further underscores the critical role that *Bombella* plays in maintaining energetic homeostasis within bee larvae, a balance that is essential for their survival and development.

## 4. Discussion

Acetamiprid, with its low toxicity and broad-spectrum insecticidal advantages, has been extensively utilized as one of the few neonicotinoid insecticides approved for use in the EU and worldwide for agricultural pest control. Although acetamiprid offers considerable advantages, studies have indicated potential impacts on non-target insects such as honeybees. It is our contention that, alongside examining the direct physiological impact of pesticides on bee lifespan and physiology, researching the potential sublethal effects of these chemicals is also of great importance to comprehensively evaluate the ecological effects of acetamiprid. We conducted a thorough investigation into its influence on the gut microbial communities of adults and larvae at environmentally relevant concentrations. By integrating metabolomic data, we aim to provide a more holistic understanding of the complex interactions between acetamiprid and the gut microbiota, as well as the metabolic processes, in bees.

The results of our study revealed that exposure to both high (25 mg/L) and low (5 mg/L) doses of acetamiprid significantly altered the gut microbial structure in both adults and larvae, causing disruptions in the honeybee gut microbiota compared to the control group (0 mg/L). Treatment with acetamiprid did not significantly alter the alpha diversity of adult bees, but it did significantly alter the beta diversity, causing significant changes in the three species *Commensalibacter*, *Bifidobacterium*, and *Gilliamella.* The relative abundance of *Commensalibacter* increased significantly in the acetamiprid groups, from an average relative abundance of 0.92% in the control group to 39.24% (5 mg/L) and 43.90% (25 mg/L), respectively. *Commensalibacter* is a widespread insect symbiont with strict ecological niches in the honeybee gut, and each species in the genus contributes to the host physiology in a specific way [51,52]. Research has shown a strong correlation between *Commensalibacter* abundance and bee health. For instance, its levels rise in bees infected with Varroa and in long-lived winter bees, contributing positively to hive survival [47,53]. Among other hosts, *C. intestini* was reported to be involved in modulating *Drosophila* immunity and suppressing the proliferation of *Gluconobacter morbifer* by competition [54]. An increased abundance of *Commensalibacter* was correlated with a longer host lifespan in *Speyeria mormonia* butterflies [55]. Our study revealed that acetamiprid exposure in bees led to a significant increase in *Commensalibacter*, potentially due to its involvement in acetamiprid metabolism and the utilization of nitrogenous byproducts, highlighting its importance in bee health and the pesticide response [56]. This leads to the possibility that *Commensalibacter* may play a role in immunometabolism in adult worker bees faced with exposure to stressors such as acetamiprid, as well as further evidence that changes in its relative abundance are tightly correlated with bee health. The *Bifidobacterium* and *Gilliamella* abundance decreased significantly in the 5 mg/L treatment group, and a decreasing trend was demonstrated in the 25 mg/L treatment group. *Bifidobacterium* and *Gilliamella* are both core phylotypes of honeybee workers and are beneficial bacteria, and a decrease in the relative abundance of these two genera may result in increased susceptibility to pathogens and a partial immune-metabolic deficit in honeybees. *Bifidobacterium* has been shown to inhibit the growth of other microorganisms in culture [57], and it has the ability to not only decompose food but also to inhibit the growth of other microorganisms in culture. At the same time, it has the ability to break down not only semifibrous and pectin substances in food but also a variety of antioxidant enzymes and respiratory chain enzymes, which allows it to participate in oxidative metabolic processes [58]. *Gilliamella* is closely related to the intestinal metabolism of honeybees and has the ability to break down pollen cell walls and ferment substances such as mannose, arabinose, xylose, and rhamnose [35]. A decrease in these two bacteria may be one of the important reasons for the weakening of carbon and water metabolism and immunity in honeybees, while the increase in *Commensalibacter* may be a result of compensation for the decrease in the two core bacteria and maintaining the dynamic balance of the intestinal tract. Thus, disruptions in the gut microbiomes of worker bees may be one reason for the impaired immune metabolism of workers after exposure to acetamiprid, and they may be responsible for the increased risk of intestinal diseases and opportunistic pathogen infections in adults [59], as well as one of the reasons for the shortened lifespans of adults [25]. In the study conducted by Wensu Han, they observed no significant impact of acetamiprid exposure on the gut microbiome of *Apis cerana* [60]. We hypothesize that this discrepancy with our findings could stem from differences in the mode and dose of exposure employed in the two studies. Specifically, the dose used in their study was substantially lower than that utilized in our experiments. Furthermore, the literature suggests that the effects of pesticides on gut microbiomes can differ significantly among bee species, which may account for the distinct results observed in our study compared to others [61].

It is not only honeybee worker bees that are exposed to acetamiprid during hive life and foraging, but, since honeybee larvae also have pollen and honey in their diet, there is also the potential for them to be orally exposed to acetamiprid. Therefore, our study analyzed the changes in the gut microbiomes of honeybee larvae after exposure to 5 mg/L and 25 mg/L concentrations of acetamiprid. We found that, regardless of whether the larvae were treated with 5 mg/L or 25 mg/L of acetamiprid, their gut microbiomes exhibited extensive and significant disruption compared to the normal gut microbiome of the control group. The dominant bacteria in the control group of the present study, namely *Proteobacteria* (70.10%), *Firmicutes* (21.70%), and *Cyanobacteria* (4.79%), were generally in agreement with the results of previous studies on the larval gut microbiota [39,43,62]. In the 5 mg/L and 25 mg/L treatment groups, notable shifts occurred in the relative abundances of *Proteobacteria* and *Cyanobacteria*. Specifically, the relative abundance of *Proteobacteria* decreased by 24.52% and 54.86%, respectively, while that of *Cyanobacteria* increased by 17.50% and 59.73%. It is our hypothesis that the extensive and pronounced disorder observed within the larval microbial community, accompanied by significant variations in the relative abundance of many of these microbial species, is a consequence of major shifts in the dominant species of *Proteobacteria* and *Cyanobacteria*. This results in an overall attempt by the intestinal microbiota to reach a new equilibrium state. Meanwhile, the gut microbiota of honeybee larvae is extremely susceptible to environmental influences and can undergo drastic changes [63,64]. The presence of certain seemingly atypical bacteria in larvae is likely due to the natural process of acquiring microbiota from the hive environment through the interaction between larvae and worker bees [65]. We further analyzed the differences in the gut microbiome at the larval genus level, and the effect of acetamiprid was also significant at the genus level, with 11 of the top 15 abundance groups showing significant changes. *Bombella* was the most abundant group at the genus level in the control group (67.89%), with a decreasing tendency in the 5 mg/L treatment and a significant change in the 25 mg/L treatment. A related study showed that *Bombella* was the dominant bacterial colony in 5-day-old honeybee larvae, and it had a positive effect on larval development. Additionally, a *Bombella* intervention enhanced the pupation rate, emergence rate, and overall survival rate [66]. In contrast, a previous study on the effects of acetamiprid on *Apis mellifera* revealed that a concentration of 5 mg/L of acetamiprid had a disruptive influence on honeybee development, including their initial weight and pupation rate, while the larval emergence rate was significantly reduced at a concentration of 25 mg/L [25]. Pesticides that indirectly disrupt the honeybee gut microbiota by impairing host health may require longer exposure times to cause microbiota disruption, as they accumulate in the host over time. Conversely, pesticides with direct effects on the gut microbiota can rapidly cause severe disruption within a shorter period, thereby compromising host health [60]. We believe that acetamiprid’s effect on larvae is more likely a direct effect on *Bombella*, resulting in various adverse effects on the larvae. *Bombella*, a bacterium that is crucial for regulating larval nutrition in holometabolous insects [67,68,69], has emerged as a key contributor to nutritional stress management in honeybees. It supplies all essential amino acids that are vital for the growth and development of honeybee larvae [50]. The massive loss of *Bombella*, potentially caused by acetamiprid, may lead to a significant decrease in larval pupation, emergence, and survival rates due to the combined stress of inadequate nutrient uptake (especially amino acids) and other effects of the pesticide. Researchers have found that *Bombella* supplementation can alleviate some negative effects of antibiotics, restoring the health of honeybee larvae [66]. This research indirectly reinforces our view that the numerous adverse effects of acetamiprid treatment on larvae are closely linked to the significant reduction in *Bombella* in the gut.

To further delve into this area, we conducted a comprehensive analysis by correlating the relative abundance of the bacterial genera in honeybee larvae following acetamiprid exposure with the differential metabolites identified in these treated larvae. Our correlation analysis uncovered significant positive associations between numerous differential bacterial species and metabolites, including p-xylene, vanillin, nicotinic acid, and hippurate. These metabolites are intricately involved in metabolic pathways such as nicotinic acid and nicotinamide metabolism, phenylalanine metabolism, and the tricarboxylic acid (TCA) energy cycle. This observed correlation may be attributed to the impact of neonicotinoid pesticides, such as acetamiprid, on the energy metabolism of honeybees. The altered gut microbiota, specifically these bacterial populations, may assist the organism in mitigating the adverse stress induced by acetamiprid exposure. This mitigation could occur through mechanisms that enhance energy metabolism and modulate immune responses, potentially helping honeybees to cope with the harmful effects of the pesticide. Based on the analysis, *Bombella* emerges as a key indicator species within the gut microbiota, signaling the potential health risks posed by acetamiprid exposure to honeybee larvae. Notably, the significant correlation between *Bombella* and a variety of amino acids aligns with prior research, reinforcing the fact that *Bombella* possesses enzymes capable of synthesizing all essential amino acids necessary for honeybee nutrition and larval growth [50]. Furthermore, the strong link between *Bombella* and the tryptophan metabolic pathway suggests that a notable decrease in *Bombella* abundance may have far-reaching consequences, potentially impeding the neurodevelopment of the larval brain [51]. This underscores the crucial role that *Bombella* plays in maintaining the overall health and well-being of honeybee larvae [70].

After conducting this study, we discovered that the gut microbial communities of both worker bees and larvae underwent significant changes when exposed to acetamiprid at concentrations of 5 and 25 mg/L. It is noteworthy that some of these changes were more evident at the lower dose, suggesting a potential nonlinear relationship between the acetamiprid concentration and the gut microbiota of bees. This observation aligns with some existing research on neonicotinoids, which has found that low concentrations of pesticides can elicit significant disturbances, whereas higher concentrations may not exhibit notable effects [71,72]. Concurrently, recent studies have demonstrated that bees are not only susceptible to insecticides but also to specific adjuvants present in pesticides, including organosilicone surfactants, nonylphenol polyethoxylates, and the solvent N-methyl-2-pyrrolidone (NMP) [73]. In light of this, further research is required in the future to gain a deeper understanding of the effects of the compounds present in acetamiprid or other pesticides on the gut microbiota and other relevant aspects.

## 5. Conclusions

This study revealed that acetamiprid exerts a considerable disruptive impact on the intestinal flora of adult and larval honeybees. Exposure to acetamiprid results in a significant decrease in the relative abundance of some core gut metabolites in adult bees, accompanied by a marked increase in *Commensalibacter*. For larvae, the changes in the gut microbiota induced by acetamiprid exposure are even more extensive and pronounced. Notably, the significant decrease in the abundance of *Bombella*, a crucial nutritional symbiont for larval bees, could be one of the primary reasons behind the previously observed reductions in the larval hatching and survival rates under acetamiprid exposure. Furthermore, the significant correlations between the differential metabolites in larvae and various differential microbial taxa further underscore the intimate connection between the gut microbiota and host health. In particular, the effects of acetamiprid on larval energy metabolism and neuroregulation reflect the vital function of the gut microbiota in the development and detoxification processes of bees. In the future, it is necessary to further validate the specific functional roles of these differential microbiota in bees and comprehensively summarize the disruptive effects of other insecticides and compounds contained in pesticides on bees’ gut microbial communities, in order to conduct a comprehensive assessment of the potential risks posed by insecticides to bees.

## Figures and Tables

**Table 1:** Differential metabolite aggregation and correlation in metabolic pathways.

**Pathway**	**Classification**	**Compound Name**	**Relevance**
Tryptophan metabolism	Neurometabolic	Arachidonic acid; 5-hydroxyindole-3-acetic acid	+
Phenylalanine metabolism	Energy Metabolism	L-kynurenine; 5-hiaa; 5-hydroxyindoleacetate Vanillin; N-acetyl-l-phenylalanine; L-tyrosine	+ −
Purine metabolism	Energy Metabolism	Uric acid; guanine; guanosine; inosine 2′-deoxyguanosine; xanthine; xanthosine	+ −
Tyrosine metabolism	Neurometabolic	Tyrosol; L-tyrosine	−
Caffeine metabolism	Neurometabolic	Xanthine; xanthosine; theophylline; caffeine	−

The plus sign (+) denotes a positive correlation, whereas the minus sign (−) indicates a negative correlation.

## Data Availability

Data will be made available on request.
